# Effect of topical papaverine on parathyroid function preservation after total thyroidectomy: a retrospective cohort study

**DOI:** 10.3389/fendo.2025.1675722

**Published:** 2025-11-20

**Authors:** Jie Li, Binbin Li, Binxin Hou, Yinqin Wang, Xiaonuo Shi, Ying Yan, Wenhao Duan, Shouhua Zheng

**Affiliations:** 1 Department of Thyroid Surgery, The First Affiliated Hospital of Zhengzhou University, Zhengzhou, China; 2 School of Medicine, Lanzhou University, Lanzhou, China; 3 Department of General Surgery, Zhengzhou Central Hospital, Zhengzhou, China

**Keywords:** papaverine, total thyroidectomy, parathyroid function, microcirculation, thyroid gland

## Abstract

**Objective:**

To evaluate the effectiveness of locally applied papaverine in preventing hypoparathyroidism after total thyroidectomy and its impact on parathyroid hormone (PTH) recovery.

**Study design:**

Retrospective cohort study.

**Setting:**

The First Affiliated Hospital of Zhengzhou University.

**Methods:**

This single-center retrospective cohort study involved 226 patients who underwent total thyroidectomy at the First Affiliated Hospital of Zhengzhou University from March 2023 to March 2025. The incidence of hypoparathyroidism on postoperative day 1 and the PTH recovery rate within 30 days afterward are compared between patients who received papaverine and those who did not. Data analysis uses the independent samples t-test, the Mann-Whitney U test, and the χ² test.

**Results:**

Patients are divided into two groups: those who received intraoperative papaverine (n = 106) and a control group (n = 120). On postoperative day 1, hypoparathyroidism occurs in 40% of the papaverine group and 53% of the control group, respectively. Postoperative PTH recovery rates gradually increase over time, with significantly higher rates in the papaverine group compared to the control group on days 3, 5, and 30.

**Conclusion:**

Applying papaverine directly during surgery effectively lowers the risk of early hypoparathyroidism after total thyroidectomy while promoting faster PTH recovery. This method is simple, easy to perform, and has a high safety profile. Future multicenter randomized controlled trials should be conducted to confirm long-term effectiveness and refine the dosing protocol.

## Introduction

Total thyroidectomy is the standard treatment for thyroid cancer and complex benign thyroid diseases (1). Postoperatively, total thyroidectomy can cause hypocalcemia and hypoparathyroidism (HP), which are significant complications linked to this surgery (2–4), occurring at rates as high as 20%-50% (5). These complications increase the risk of developing hand-foot spasms (carpopedal spasms), arrhythmias, and osteoporosis in patients (6, 7). Current protective measures, such as parathyroid autotransplantation or postoperative calcium supplementation, face limitations due to low graft survival rates and long-term medication side effects (8–11). This situation calls for urgent improvements in intraoperative interventions.

The intraoperative protection of parathyroid function depends on maintaining the blood supply (12, 13). The microvessels of the parathyroids are anatomically fragile and susceptible to traction or thermal injury during surgery, which can cause spastic ischemia (14, 15). Local vasodilation may be crucial for improving perfusion. Papaverine, a phosphodiesterase inhibitor, induces vasodilation by increasing intracellular cyclic adenosine monophosphate (cAMP) levels (16, 17). It has been successfully used for microcirculatory protection in cardiovascular and neurosurgery (18–20). However, evidence for its use in thyroid surgery is somewhat limited. Through retrospective comparative analysis, this study aims to assess the effect of intraoperative topical application of papaverine on the incidence of hypocalcemia and HP after total thyroidectomy, as well as on PTH recovery. It also examines potential connections with microcirculatory improvement, offering new insights for refining intraoperative parathyroid protection strategies.

## Materials and methods

### Study population

This retrospective cohort study includes data from 280 patients who underwent total thyroidectomy at the Department of Thyroid Surgery, First Affiliated Hospital of Zhengzhou University, from March 2023 to March 2025.

- Papaverine group (n=106): March 2024–March 2025 (routine topical papaverine irrigation).
- Control group (n=120): March 2023–March 2024 (standard saline irrigation).

The allocation was determined solely by the surgery date, reflecting an institutional protocol update in March 2024 to include papaverine for parathyroid protection. All operations were performed by a fixed senior surgical team (annual thyroidectomy volume >100 cases) that adhered to contemporary technical standards. Notably, surgeons’ technical competence had reached a plateau phase before study initiation, and no innovative techniques/devices were introduced during the study period. The clinical data collected include gender, age, pathology, Lymph nodes removed, preoperative PTH and calcium levels, serum calcium on postoperative day 1, and PTH levels on postoperative days 1, 3, 5, and 30. All data were extracted from the medical records system of the First Affiliated Hospital of Zhengzhou University.

#### Inclusion criteria

- Age between 18 and 75 years
- Normal preoperative PTH (15–65 pg/mL) and serum calcium (2.0–2.7 mmol/L) levels
- Total thyroidectomy for thyroid disease performed at our hospital using a standardised technique: capsular dissection approach. For malignant cases, this includes bilateral central neck dissection (level VI)
- All surgeries carried out by the same surgical team in the Department of Thyroid Surgery
- Complete clinical and follow-up data available

#### Exclusion criteria

- Intraoperative parathyroid compromise: incidental parathyroid resection (confirmed histologically), parathyroid autotransplantation
- Oncologic considerations (malignant cases only): lateral cervical lymph node metastasis, distant metastasis, locally advanced tumours requiring extended resection
- Surgical outcomes: operative time over 3 hours; significant blood loss (>300 mL or requiring transfusion)
- Medical history contraindications: previous neck surgery, coagulation dysfunction, permanent hypoparathyroidism, parathyroid disease, metabolic bone disease, renal insufficiency, or long-term use of calcium/vitamin D supplements

This study has been approved by the Ethics Committee of the First Affiliated Hospital of Zhengzhou University (Approval No.: 2025-KY-0888). Informed consent was obtained from patients or their families.

### Surgical procedure

All procedures were carried out by the same experienced surgical team (>100 cases per surgeon per year) following institutional protocols.

#### Anatomical extent

##### Benign disease

Total thyroidectomy with capsular dissection, preserving the parathyroids *in situ*. The central compartment was not routinely explored. Malignancy: Total thyroidectomy with systematic bilateral central lymph node dissection (level VI) regardless of preoperative nodal status. Patients undergoing central dissection were retained in the analysis to reflect real-world surgical management and ensure clinical applicability, with baseline pathological types (benign vs malignant) balanced between groups (**Table 1**).

**Table 1:** Hypocalcemia management protocol after thyroidectomy.

PTH (pg/mL)	Normal ca^2+^; (2.0≤Ca<2.7 mmol/L)	Mild hypocalcemia (1.8≤Ca<2.0 mmol/L)	Severe hypocalcemia (ca<1.8 mmol/L)
PTH ≥15	Observation	Oral: Calcium-D_3–_1200 mg QD IV: Calcium gluconate 3g/day	Oral: Calcium-D_3–_1200 mg QD IV: Calcium gluconate 6g/day
10≤PTH<15	Oral: Calcium-D_3–_1200 mg QD Alfacalcidol 0.25 μg QD	Oral: Calcium-D_3–_1200 mg QD Alfacalcidol 0.25 μg QD IV: Calcium gluconate 3g/day	Oral: Calcium-D_3–_1200 mg QD Alfacalcidol 0.25 μg QD IV: Calcium gluconate 6g/day
5≤PTH<10	Oral: Calcium-D_3–_1200 mg BID Alfacalcidol 0.25 μg QD	Oral: Calcium-D_3–_1200 mg BID Alfacalcidol 0.25 μg BID IV: Calcium gluconate 6g/day	Oral: Calcium-D_3–_1200 mg BID Alfacalcidol 0.25 μg BID IV: Calcium gluconate 9g/day
PTH<5	Oral: Calcium-D_3–_1200 mg BID Alfacalcidol 0.25 μg BID IV: Calcium gluconate 3g/day	Oral: Calcium-D_3–_1200 mg TID Alfacalcidol 0.25 μg BID IV: Calcium gluconate 6g/day	Oral: Calcium-D_3–_1200 mg TID Alfacalcidol 0.25 μg TID IV: Calcium gluconate 9g/day

a: Calcium carbonate-Vitamin D3 tablets: Each tablet contains 600 mg elemental calcium (as carbonate) + 125 IU cholecalciferol (Vitamin D3) (Jintai Pharma, Hainan, China).

b: Alfacalcidol tablets: 0.25 μg per tablet (equivalent to 10 IU Vitamin D3 biological activity).

c: IV calcium gluconate protocol: 3 g (three 10 mL vials of 10% calcium gluconate solution, 1 g/vial) diluted in 100 mL of 5% dextrose or 0.9% sodium chloride; Total elemental calcium = 270 mg (2.25 mmol per 1 g gluconate), infused over ≥30 minutes.

##### Parathyroid identification and preservation:

###### Minimal exposure principle

Parathyroids were identified through a combination of nanocarbon negative imaging (1 mL intra-thyroidal injection before dissection) and direct visual assessment by the operating surgeon. Identification criteria included: Typical anatomical location within parathyroid zones; Characteristic tan-to-yellow colour distinct from surrounding fat; Oval or flattened shape with a smooth surface; Clear vascular pedicle seen with nanocarbon contrast.

All glands were preserved *in situ* without excessive dissection or deliberate skeletonisation of feeding vessels to prevent iatrogenic injury. This approach aligns with evidence from a randomised trial showing that minimising manipulation reduces transient hypoparathyroidism compared to traditional vascular dissection (21).

#### Energy device and hemostasis

##### Primary device

Harmonic Focus^®^ scalpel (Ethicon GEN11), with the acknowledged limitation of residual thermal risk to glands adhering to the thyroid capsule.

### Safety monitoring protocol

All patients receiving intraoperative papaverine were subjected to protocolized safety monitoring. Intraoperative hemodynamics were tracked every 5 minutes using automated non-invasive blood pressure (NIBP) devices, with predefined clinical thresholds: significant hypotension was defined as a mean arterial pressure <65 mmHg sustained for more than 3 minutes, and tachycardia as a heart rate >120 bpm. Postoperative monitoring included standardized assessments every 8 hours during the first 72 hours, screening for allergic reactions (such as new rash, bronchospasm, or oxygen saturation decline of ≥5%) and local complications (including hematoma formation, with ultrasound quantification if drainage output exceeded 100 mL/24h). Delayed adverse events were recorded through structured telephone interviews at postoperative day 30. To attribute events to papaverine, all three criteria had to be met: (1) a clear temporal relationship (≤72 hours after administration), (2) biological plausibility based on established papaverine pharmacology, and (3) no alternative causes explaining the event.

### Observation indicators

Exposure Variable: Intraoperative application of papaverine:

- The surgical site was locally irrigated with a solution containing 30 mg of papaverine. This solution was prepared immediately before use by diluting 1 ml of papaverine hydrochloride injection (30 mg/ml) with 1 ml of physiological saline, resulting in a final concentration of 15 mg/ml.
- Timing and Technique: Solution (~2 mL total volume) was irrigated locally over the thyroid bed and parathyroid tissues after complete thyroidectomy and hemostasis, before wound closure. The solution was left *in situ* to undergo natural tissue absorption and dilution with serous exudate during the final minutes of surgery.
- Control Group: An identical volume of saline (2 mL) was irrigated and retained *in situ* using the same protocol.
- Outcome Variables:
- hypoparathyroidism: Defined as serum parathyroid hormone (PTH) < 15 pg/mL on the first postoperative measurement and/or serum total calcium <2.0 mmol/L. (within 24 hours)
- Parathyroid function recovery rate: Defined as PTH ≥ 15 pg/mL. Serum PTH is measured in the morning on postoperative days 3, 5, and 30, and the proportion of patients achieving recovery is calculated.

Confounding Variables: age, gender, pathology, preoperative PTH, operation time, blood loss, and preoperative serum calcium.

### Standardized postoperative calcium and vitamin D management protocol

All patients received standardized postoperative calcium management to prevent bias from supplementation. A strict non-prophylaxis policy was enforced: no routine calcium or vitamin D supplements were given. Serum total calcium was measured within 24 hours after surgery, with repeat testing based on clinical signs, and nursing assessments for hypocalcemia symptoms (numbness, paresthesia, muscle twitching, Chvostek/Trousseau signs) were performed every 8 hours. Intervention was initiated if any of the following occurred: (1) biochemical hypocalcemia (serum calcium <2.0 mmol/L or PTH <15 pg/mL), or (2) clinical hypocalcemia symptoms requiring immediate IV calcium gluconate 3g, regardless of lab results. Stratified replacement protocols were applied based on 24-hour postoperative PTH and calcium levels (**Table 1**).

Transition to oral-only therapy was allowed when: serum calcium was ≥2.0 mmol/L for 48 hours without IV supplementation; neuromuscular irritability was absent (Chvostek/Trousseau sign negative); and the patient demonstrated competency in oral medication scheduling.

### Statistical methods

Data analysis was conducted with SPSS 27.0. Continuous variables were tested for normality using the Shapiro-Wilk test and analysed with either the independent samples t-test (parametric) or the Mann-Whitney U test (non-parametric). Normally distributed data are presented as mean ± standard deviation (Mean ± SD), non-normally distributed data as median (interquartile range) [M (IQR)], and categorical variables as frequency (percentage) [n (%)]. Group comparisons used the χ² test or Fisher’s exact test for categorical outcomes. The robustness of significant associations was assessed with E-values, which estimate the minimum strength of unmeasured confounding needed to explain away observed effects. For risk ratios (RR) < 1 (e.g., hypocalcemia), E-values were calculated directly; for RR > 1 (e.g., PTH recovery), 1/RR was used before calculating the E-value. All tests were two-tailed, with P < 0.05 indicating statistical significance.

### Data availability statement

Access to original data is restricted to protect patient privacy. De-identified datasets are available from the corresponding author upon reasonable request, pending approval by the Ethics Committee of the First Affiliated Hospital of Zhengzhou University (Ref: 2025-KY-0888).

## Results

### Study cohort

**Figure 1:**
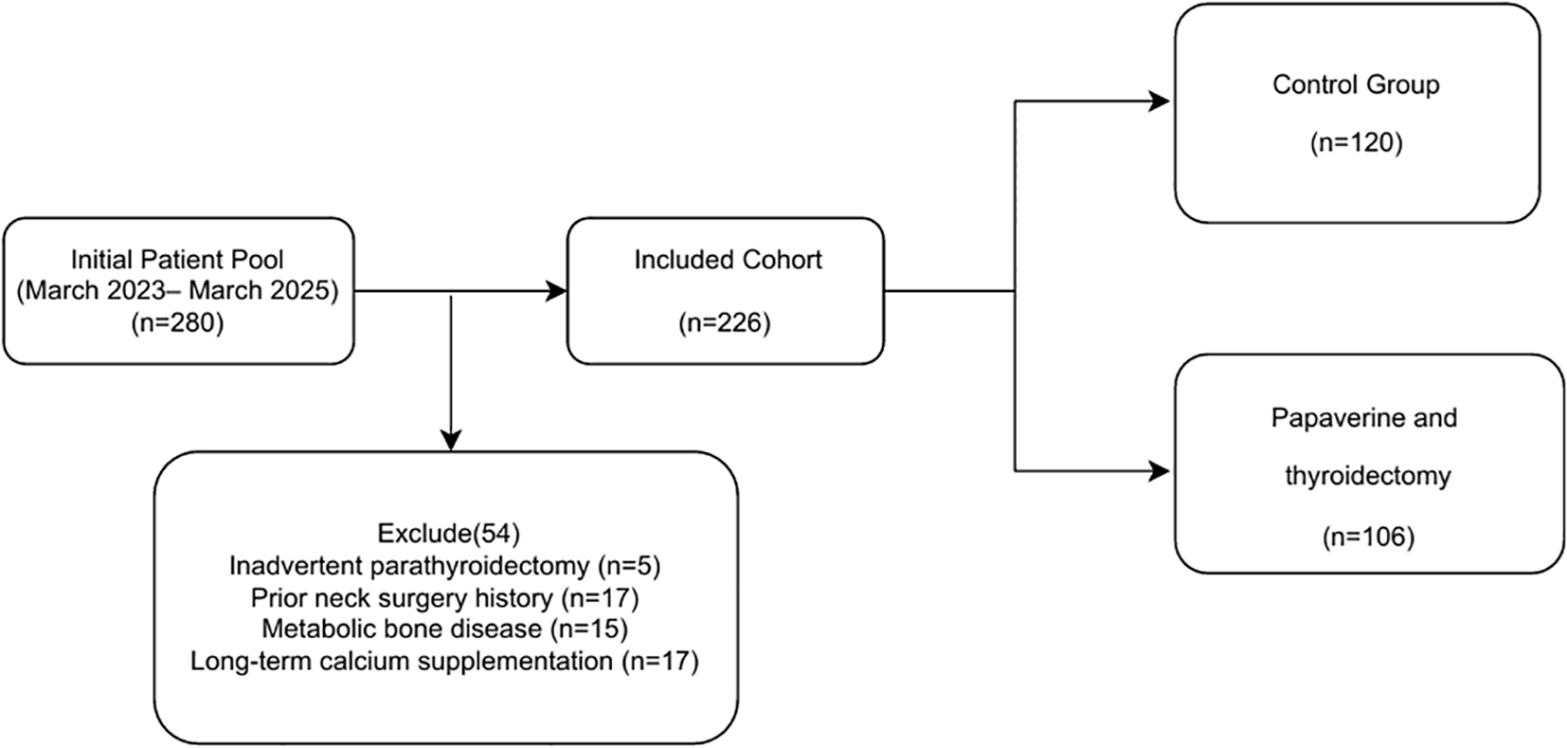
Flowchart of patient inclusion.

From March 2023 to March 2025, a total of 280 patients who underwent total thyroidectomy were screened at the Department of Thyroid Surgery at the First Affiliated Hospital of Zhengzhou University. After excluding cases of inadvertent parathyroidectomy (n = 5), previous neck surgery (n = 17), metabolic bone disease (n = 15), and long-term calcium supplementation (n = 17), 226 patients were ultimately included in the study. The detailed screening process is shown in **Figure 1**. Based on the intraoperative use of topical papaverine, patients were divided into two groups: the papaverine group (n = 106) and the control group (n = 120). Baseline characteristics, including age, sex, pathology, operative time, blood loss, and preoperative PTH/calcium levels, were well balanced between the papaverine and control groups (all P > 0.05, **Table 2**). This indicates that the natural experiment design effectively minimised selection bias, allowing for a direct comparison of unadjusted outcomes.

**Table 2:** Baseline characteristics of the papaverine and control groups.

Characteristic	Papaverine group (n=106)	Control group (n=120)	P
Age	47 (20–70)	46 (19–74)	0.50
Sex			
Female	83 (78%)	96 (80%)	0.75
Male	23 (22%)	24 (20%)	0.75
Pathology			
Benign	52 (49%)	58 (48%)	0.89
Malignant Lymph nodes removed	54 (51%) 5 (3–9)	62 (52%) 6 (4–10)	0.89 0.52
Operation Time (min)	73.7 (53-98)	73.4 (55–95)	0.89
Blood Loss (ml)	11.2 (5–30)	11.9 (5-30)	0.09
Preoperative PTH (pg/mL)	37.8 (15.4–64.3)	32.4 (15.0–64.6)	0.11
Preoperative Calcium (mmol/L)	2.30 ± 0.1	2.28 ± 0.1	0.14

PTH, Parathyroid Hormone; Lymph node count reflects central neck dissection extent in malignant cases only (n=116, 54 papaverine, 62 control); benign cases had no dissection.

### Postoperative outcomes

Incidence of Hypoparathyroidism (HP) on Postoperative Day 1:The HP rate is 40% in the papaverine group, significantly lower than the 53% in the control group (RR = 0.74, 95% CI 0.56–0.98, P = 0.04).To evaluate the robustness of these associations, we calculated E-values, which estimate the minimum strength of unmeasured confounding required to explain away the observed effects (22). For HP, the E-value was 1.99, meaning nearly doubling the risk would be necessary. The value exceeded the conventional threshold by 1.5, confirming the stability of the research findings.

### Dynamic analysis of PTH recovery

PTH recovery in both groups shows consistent improvement over time (**Table 3**):

**Table 3:** Comparison of postoperative outcomes in the cohort.

Outcome measures	Papaverine group (n=106)	Control group (n=120)	RR (95% CI)	P	E-value
Hypoparathyroidism	42 (40%)	63 (53%)	0.74 (0.56-0.98)	0.04	1.99
PTH Recovery Rate at Day 3	74 (70%)	64 (53%)	1.31 (1.06–1.62)	0.01	1.73
PTH Recovery Rate at Day 5	79 (75%)	64 (53%)	1.42 (1.15–1.74)	<0.001	2.05
PTH Recovery Rate at Day 30	97 (92%)	98 (82%)	1.12 (1.02–1.24)	0.03	1.36

E-value = RR + √[RR×(RR−1)] (for RR<1) or 1/RR + √[(1/RR)×((1/RR)−1)] (for RR>1).

- Postoperative Day 3: The PTH recovery rate in the papaverine group is 17% higher than in the control group (RR = 1.31, 95% CI 1.06–1.62, P = 0.01).
- Postoperative Day 5: The recovery rate difference increases to 22% (RR = 1.42, 95% CI 1.15–1.74, P < 0.001).
- Postoperative Day 30: The papaverine group maintains its advantage (RR = 1.12, 95% CI 1.02–1.24, P = 0.03).

E-values for PTH recovery were derived by inverting the RR (1/RR) to align with the protective effect framework (22). The Day 30 E-value (1.36), while slightly below the 1.5 threshold, still indicates moderate robustness considering the significant P-value.

### Safety outcomes

Continuous monitoring via NIBP showed no instances of significant hypotension (no MAP below 65 mmHg lasting more than 3 minutes) or sustained tachycardia. Over 72-hour assessments and a 30-day telephone follow-up (100% completed), no papaverine-related events were observed, evidenced by: zero allergic reactions, no ultrasound-confirmed hematomas, and no late complications meeting pharmacologic attribution criteria.

## Discussion

This retrospective comparative analysis demonstrates that the intraoperative topical application of papaverine effectively reduces the occurrence of hypocalcemia and early HP after total thyroidectomy, while also aiding quicker PTH recovery. This finding is mechanistically plausible because of papaverine’s known microcirculatory-enhancing properties, which have been documented in other ischemic-vulnerable tissues (such as vascular and neuronal systems) (23–25). The underlying mechanism of our observed benefit likely involves papaverine-induced dilation of parathyroid microvasculature, improved local blood flow, and a subsequent reduction in intraoperative ischemic injury.

**Figure 2:**
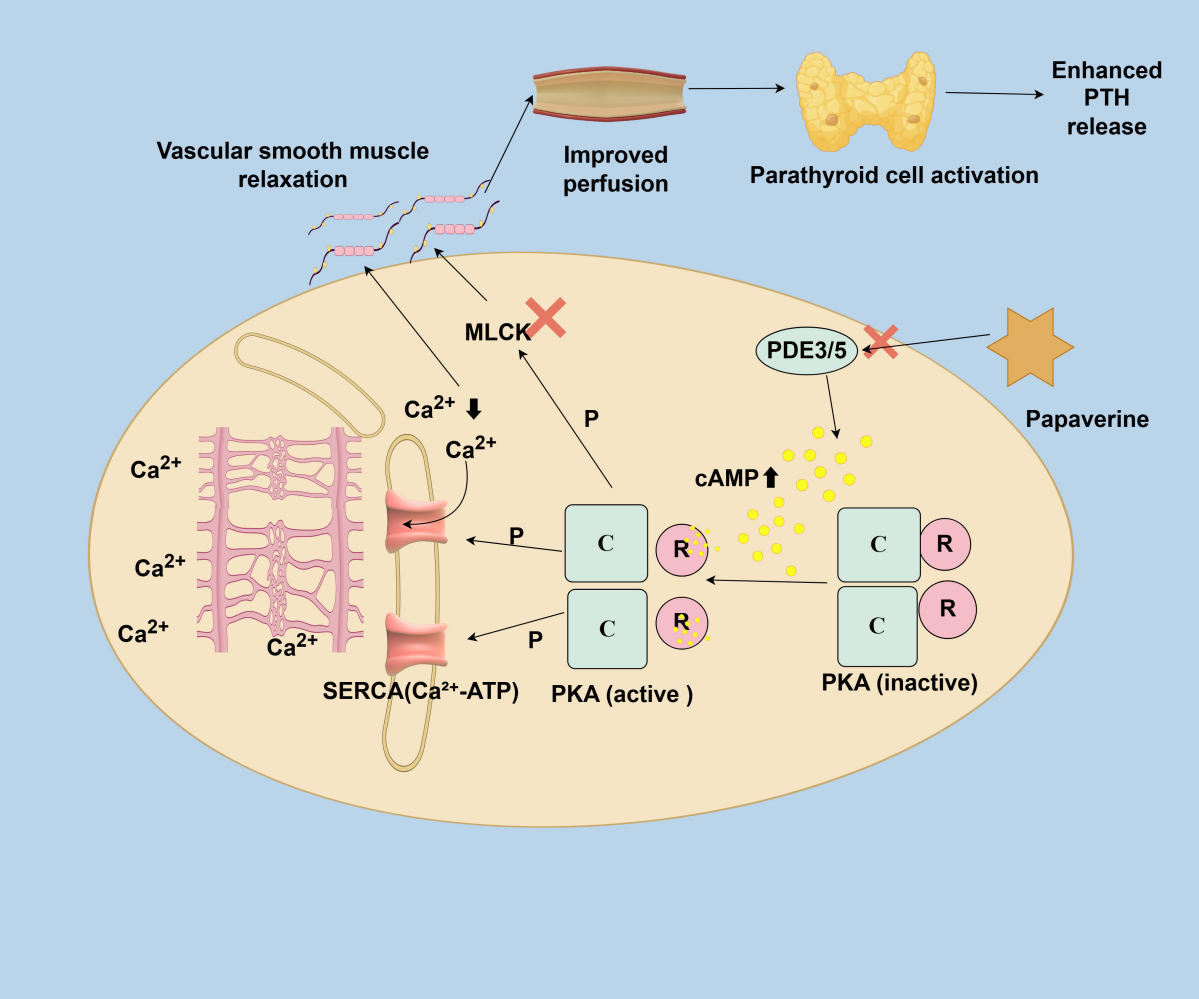
Mechanism of papaverine-induced vasodilation in parathyroid microvessels via cAMP-PKA pathway.

Regarding the mechanism of action (**Figure 2**), topical spray administration of papaverine solution penetrates the microvascular smooth muscle cells of the parathyroid glands. By inhibiting phosphodiesterases (PDE3 and PDE5), it significantly raises intracellular cyclic adenosine monophosphate (cAMP) levels. The accumulation of cAMP activates protein kinase A (PKA), which then phosphorylates two key calcium-regulatory proteins: (1) Sarcoplasmic/Endoplasmic Reticulum Ca^2+^-ATPase (SERCA), enhancing its activity, speeds up the reabsorption of cytosolic calcium ions into the sarcoplasmic reticulum, and reduces cytosolic Ca^2+^ concentration; (2) Myosin Light Chain Kinase (MLCK), decreasing its activity, which lowers phosphorylation of contractile proteins and promotes their disassembly. The blood supply to the parathyroid is fragile and prone to spastic ischemia during surgery due to manipulation and thermal injury. Topical application of papaverine may reverse this microvascular constriction, ensuring proper oxygenation and meeting the metabolic demands of the parathyroid, thus lowering the risk of postoperative functional impairment. Evidence supporting this includes the finding that PTH levels on postoperative day 1 are significantly higher in the papaverine group compared to the control group, suggesting that early hemodynamic improvements may help preserve function.

Compared to existing protective strategies, topical papaverine offers advantages in ease of application, low cost, and safety. No papaverine-related adverse reactions were observed during this study. Unlike intraoperative parathyroid autotransplantation (which prolongs surgery and carries a risk of graft failure) or postoperative high-dose calcium supplements (which may cause hypercalcemia or gastrointestinal issues), papaverine intervention better aligns with minimally invasive procedures. The persistent benefit in PTH recovery rates within the papaverine group from postoperative days 3 to 30 suggests it may help reduce the risk of permanent hypoparathyroidism; however, longer-term studies are needed to confirm this.

This study has several limitations:

- The primary limitation stems from the historical control design, introducing time-related confounding. However, the risks are mitigated by: (a) engagement of high-volume surgeons (>100 cases/year) whose performance plateaued pre-study, and (b) strict prohibition of new technologies during the trial period. Nevertheless, residual temporal biases cannot be entirely excluded.
- Retrospective design: As a retrospective cohort study, it is subject to inherent limitations such as reliance on the accuracy and completeness of medical records, potential unmeasured confounding factors despite statistical adjustments and E-value calculations, and less control over variables compared to a prospective randomised trial.
- Short-term follow-up: The 30-day observation period prevents assessment of permanent hypoparathyroidism (defined as PTH <15 pg/mL at 6 months), leaving the long-term protective effects of papaverine uncertain.
- Unmeasured anatomical and pathological factors: Thyroid gland volume and Hashimoto’s thyroiditis status—known determinants of parathyroid vulnerability—could not be analyzed due to inconsistent documentation in preoperative imaging and pathology reports (e.g., lymphocytic infiltration not routinely assessed).
- Limited power for subgroup analyses: The sample size (n=226) was too small for stratified analyses (e.g., by thyroid volume, Hashimoto’s status, Graves’ disease, Lymph Node Dissection), as such divisions would create underpowered subgroups.
- Uncontrolled thyroid function variables: Effects of Hashimoto’s status or Graves’ disease and postoperative TSH fluctuations could not be analysed due to statistical constraints and physiological variability.
- Single-centre homogeneity: All procedures were performed by a high-volume team using standardised techniques, which may limit how applicable the results are to centres with different surgical protocols.
- Fixed-dose design: Using only a single dose of papaverine (30 mg) prevents investigation of dose-response relationships. Future trials should examine optimal dosing ranges.
- Our study has inherited limitations from its proof-of-concept design: The calculated β-error risk of 35.2% signifies considerable uncertainty about the intervention’s maximum possible protection. Therefore, these positive results require confirmation in a larger, adequately powered cohort before they can be used to influence practice.

## Conclusion

This study shows that intraoperative topical irrigation with papaverine significantly reduces the risk of early hypoparathyroidism (HP) after total thyroidectomy and helps restore PTH levels. The mechanism may involve papaverine widening parathyroid microvessels and decreasing ischemia-reperfusion injury. Compared to traditional methods like autotransplantation and high-dose calcium supplementation after surgery, this approach is easier and safer, with no drug-related side effects observed. It is proposed as a potential intraoperative method to protect the parathyroid. Significantly, the sample size limits the clinical applicability in two ways. *Post-hoc* power analysis confirmed limited detection capacity (1-β=64.8%), indicating a 35.2% risk of a type II error for therapeutic effects below 43% relative risk reduction. Additionally, the indeterminacy of permanent HP is a limitation, as the small number of cases of permanent hypoparathyroidism prevents meaningful long-term analysis. However, given the constraints of a single-center retrospective study and fixed drug doses, future multicenter randomized controlled trials are necessary to confirm its long-term effectiveness, including rates of permanent hypoparathyroidism, and to refine dosing for personalized treatment.

## Data Availability

The raw data supporting the conclusions of this article will be made available by the authors, without undue reservation.
